# Enhancing Ligand Pose Sampling for Molecular Docking

**Published:** 2023-11-30

**Authors:** Patricia Suriana, Ron O. Dror

**Affiliations:** Department of Computer Science, Stanford University

## Abstract

Deep learning promises to dramatically improve scoring functions for molecular docking, leading to substantial advances in binding pose prediction and virtual screening. To train scoring functions—and to perform molecular docking—one must generate a set of candidate ligand binding poses. Unfortunately, the sampling protocols currently used to generate candidate poses frequently fail to produce any poses close to the correct, experimentally determined pose, unless information about the correct pose is provided. This limits the accuracy of learned scoring functions and molecular docking. Here, we describe two improved protocols for pose sampling: GLOW (auGmented sampLing with sOftened vdW potential) and a novel technique named IVES (IteratiVe Ensemble Sampling). Our benchmarking results demonstrate the effectiveness of our methods in improving the likelihood of sampling accurate poses, especially for binding pockets whose shape changes substantially when different ligands bind. This improvement is observed across both experimentally determined and AlphaFold-generated protein structures. Additionally, we present datasets of candidate ligand poses generated using our methods for each of around 5,000 protein-ligand cross-docking pairs, for training and testing scoring functions. To benefit the research community, we provide these cross-docking datasets and an open-source Python implementation of GLOW and IVES at https://github.com/drorlab/GLOW_IVES.

## 1 Introduction

Protein-ligand molecular docking, which is crucial in drug discovery and molecular modeling [Kitchen et al., 2004, Ferreira et al., 2015], predicts the three-dimensional arrangement of ligands within target protein binding sites—a task known as “ligand pose prediction.” This computational method is vital for drug candidate exploration and understanding molecular interactions. Conventional docking software relies on sampling algorithms that generate candidate ligand poses based on a given protein structure. This task is inherently difficult due to the multitude of internal conformations the ligand can adopt and the numerous possible ways it can be placed within the protein binding site. Furthermore, a good sampling algorithm must ensure that at least one generated pose closely resembles the experimentally determined “correct pose,” which is unknown to the sampling algorithm. Scoring functions then evaluate these poses, selecting candidates predicted to closely match the correct pose.

While molecular docking has traditionally relied on physics-based scoring functions, recent advances in deep learning, as indicated by studies such as [Shen et al., 2020, Francoeur et al., 2020, Shen et al., 2022, Suriana et al., 2023], have the potential to revolutionize scoring accuracy. However, the efficacy of deep learning hinges on a crucial factor: generating suitable sets of candidate ligand binding poses. Existing sampling methods often struggle in this regard, frequently failing to produce any correct poses. This challenge intensifies when the protein structure used for docking (the “docking protein structure”) significantly differs from the conformation the protein adopts when binding to the query ligand. The inability to sample correct poses creates a twofold problem. Firstly, for an effective deep learning-based scoring function, correct poses must be included in the training dataset to allow models to learn their defining characteristics. However, introducing experimentally determined correct poses, while addressing this need, presents an artificial approach that does not reflect real-world scenarios where such data is unavailable. Moreover, incorporating experimentally determined poses during training could potentially bias the deep learning model’s judgment when applied to real-world problems, where all candidate poses, including correct ones, must be generated through sampling. Secondly, the performance of molecular docking relies heavily on the sampling algorithm’s ability to consistently yield correct poses. Even with a perfect scoring function, the absence of correct poses among candidates precludes prediction of a correct pose. Hence, there is a pressing need for an enhanced, reliable sampling method capable of consistently generating accurate ligand poses.

To address this challenge, we introduce two improved pose sampling protocols: GLOW (auGmented sampLing with sOftened vdW potential) and a novel method called IVES (Iterative Ensemble Sampling). Our protocols substantially increase the likelihood of sampling correct ligand poses, even in scenarios where clashes between the ligand’s correct binding pose and the docking protein structure are likely. Importantly, our methods do not rely on information about co-determined ligand poses in the docking protein structure, making them suitable for use with unliganded or predicted protein structures, including those generated by AlphaFold [Jumper et al., 2021, Varadi et al., 2022]. Our benchmarking demonstrates that GLOW and IVES effectively enhance ligand pose sampling accuracy for both experimental and AlphaFold-generated protein structures, as measured by the percentage of successful docking cases with correct ligand poses. Additionally, IVES generates multiple protein conformations as part of its workflow, offering considerable value for enhancing geometric deep learning techniques on protein structures and bolstering the robustness of deep learning techniques to small variations around correct poses in the context of protein-ligand docking.

To encourage broader engagement and utilization within the research community, we have created carefully curated datasets containing candidate ligand poses generated using our improved sampling methods. These datasets comprise approximately 5,000 protein-ligand cross-docking pairs, serving as invaluable resources for training and evaluating scoring functions. To promote widespread access and utilization, we have made available an open-source Python implementation of GLOW and IVES, along with the newly developed cross-docking datasets. These resources can be accessed at https://github.com/drorlab/GLOW_IVES.

## 2 Related Works

**Figure 1:**
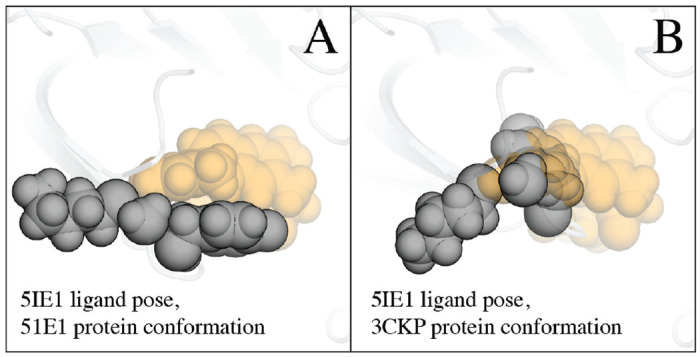
The native binding pose of a ligand often clashes with the experimentally determined structure of its target protein, especially when that structure features a different ligand. In Panel A, we observe the structure of ,0-secretase (BACE-1), a key drug target, bound to “compound 5” (PDB entry 5IE1 [Jordan et al., 2016]). Here, the ligand (depicted as orange spheres, each representing an atom) packs favorably against two protein amino acids (gray spheres) in the binding pocket with no clashes. In contrast, Panel B presents the same ligand (compound 5) in an identical geometry, but overlaid on a BACE-1 structure determined in the presence of a different ligand (PDB entry 3CKP, [Park et al., 2008]). In this case, the two amino acids (gray spheres) adopt different positions, resulting in significant clashes with the ligand atoms (orange spheres).

Numerous deep learning techniques have emerged to score candidate poses in molecular docking, traditionally relying on datasets generated through rigid protein docking, which assume fixed protein structures during sampling [Verdonk et al., 2003, Friesner et al., 2004, Allen et al., 2015, Forli et al., 2016]. For example, the CrossDock2020 model [Francoeur et al., 2020] is based on poses generated by Smina’s rigid protein docking. However, this method falls short when adjustments are needed in the docking protein structure to accommodate the correct ligand binding pose, resulting in clashes and rejection of the correct pose during rigid docking (see Figure 1). This limitation in sampling correct poses presents a significant challenge and constrains the performance of scoring functions, including those based on deep learning.

To address this, flexible protein docking methods consider protein flexibility during sampling [Jones et al., 1997, Lemmon and Meiler, 2012, Sherman et al., 2006, Miller et al., 2021]. Strategies include alternating ligand and protein sampling steps or temporarily substituting flexible residues with alanine. Some, like Schrodinger IFD-MD [Miller et al., 2021], enhance accuracy by incorporating experimentally co-determined ligand poses. Conversely, certain recent deep-learning approaches, such as Krishna et al. [2023], directly generate the protein-ligand complex. However, substantial computational resources are essential for all these methods, and flexible docking methods place an added emphasis on accurately selecting flexible residues. In practical scenarios, both flexible protein docking and deep-learning-based approaches consistently demonstrate lower accuracy compared to rigid protein docking [Ravindranath et al., 2015, Bender et al., 2021, Krishna et al., 2023].

Flexible protein docking methods and deep-learning-based approaches have been explored to enhance ligand binding pose sampling, but they bring their own limitations, including computational costs and reliance on experimental data. The introduction of GLOW and IVES seeks to tackle these challenges, providing a promising path to improve ligand pose sampling accuracy and efficiency in protein-ligand docking, which could benefit the development and evaluation of deep learning-based scoring functions.

## 3 Methods

### 3.1 Improved ligand pose sampling protocols for docking

A significant drawback of rigid protein docking is its inability to generate correct pose when it clashes with the docking protein structures. The ligand’s correct pose receives a poor score due to these clashes, leading to exceptionally high calculated van der Waals (VDW) energy values. To this end, we introduce GLOW. GLOW enhances rigid protein docking by incorporating poses generated with a softened VDW potential alongside those using a normal VDW potential.

**Figure 2:**
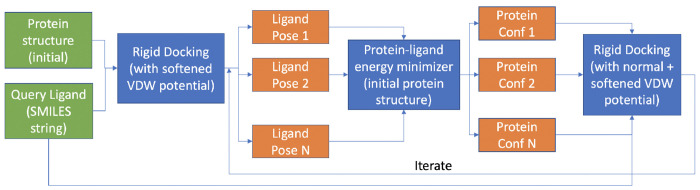
Schematic of the IVES workflow. Initially, we perform rigid protein docking using softened VDW potentials, yielding initial ligand poses. Due to this softening, some poses may clash with the docking structure. Then, we select the top N poses as “seed poses” for guiding the minimization of the input docking structure, producing an ensemble of N protein conformations. Residues within 8A of the ligand pose are allowed to move, while the rest remain fixed, including the ligand. Parallel rigid docking with normal and softened VDW potential of the input query ligand onto these N conformations follows. This process may be iterated, but typically one iteration suffices, as further iterations offer minimal improvements.

Furthermore, we present IVES, an innovative approach to enhance ligand pose sampling accuracy in protein-ligand docking (see Figure 2). IVES incorporates a combination of alternating protein-ligand pose sampling strategies inspired by flexible protein docking, all while utilizing both normal and softened VDW potentials. IVES begins with rigid protein docking, using a softened VDW potential to create initial ligand poses, allowing some clashes with the docking structure. The “seed poses,” selected from the top N poses in this initial set based on docking scores or alternative scoring functions for assessing protein-ligand docked poses, guide the minimization of the input docking structure within an 8A radius of the ligand pose, while keeping the ligand pose and other residues fixed. Subsequently, the input ligand is redocked onto these N protein conformations, employing both normal and softened VDW potentials independently for each conformation, allowing for parallelization to accelerate the process. If necessary, this step can iterate by merging poses from the prior iteration and selecting the top N ligand poses for the next iteration, although a single iteration often suffices as subsequent ones provide marginal improvements in practice.

To make our approaches accessible to a broader scientific community, we built GLOW and IVES on top of Smina [Koes et al., 2013] and OpenMM [Eastman et al., 2017], open-source software for molecular docking and protein structure minimization, respectively. In general, our approaches can be built on top of any existing software for rigid protein docking or protein minimization.

### 3.2 Datasets

We offer datasets of candidate ligand poses generated by GLOW and IVES, potentially valuable for training machine learning-based docking. Our dataset includes around 4,102 protein-ligand cross-docking pairs derived from 238 unique proteins, utilizing protein structures from the high-resolution PDBBind 2019 refined dataset [Wang et al., 2005, Liu et al., 2015]. Additionally, we provide testing datasets categorized into four groups: (1) “typical (experimental)” with 322 experimentally determined pairs (from Paggi et al. [2021]), (2) “challenging (experimental)” with 258 pairs requiring significant docking protein structure adjustments to fit the ligand (from Miller et al. [2021]), (3) “typical (AlphaFold)” features the same pairs as “typical (experimental)” but uses AlphaFold 2 protein models for docking (totaling 322 pairs), and (4) “challenging (AlphaFold)” features the same pairs as “challenging (experimental)” but uses AlphaFold 2 protein models instead of experimentally determined structures for docking (totaling 179 pairs). For more details, refer to S1.

## 4 Results

### 4.1 Evaluation of the sampling performance on the test sets

We evaluated the sampling performance of GLOW and IVES on test sets by measuring the percentage of cross-docking cases that yielded any correct pose. A correct pose was defined as having a root mean square deviation (RMSD) from the experimentally determined pose equal to or less than 2.0 A, a widely accepted practical threshold [Kontoyianni et al., 2004, Cole et al., 2005]. For reference, we included two baseline methods: (1) “Default,” representing typical docking scenarios, generating a maximum of 20 poses [Francoeur et al., 2020]; (2) “Default, max poses,” allowing the maximum number of poses, representing the upper limit of the docking protocol. For GLOW, we enabled the generation of as many poses as possible. IVES produced poses using 20 protein conformations in a single iteration and generated a maximum of 300 poses for each protein conformation. Both GLOW and IVES are implemented on Smina. For consistency, we used Smina for the baseline methods as well. Additional settings details can be found in S2.

**Figure 3:**
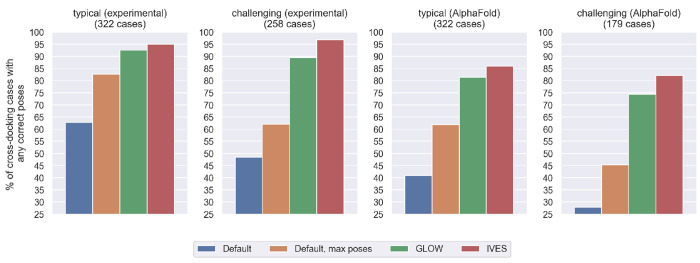
Sampling performance of GLOW and IVES on test sets, measured by the percentage of cross-docking cases with any correct pose. GLOW (green) and IVES (red) consistently outperform the baseline methods “Default” (blue) and “Default, max poses” (orange), especially for the challenging and AlphaFold benchmarks where the protein structure undergoes substantial conformational changes upon binding to the ligand, differing from the structure employed during the docking process.

Overall, GLOW and IVES consistently outperformed baseline methods, especially in challenging and AlphaFold benchmarks where the protein structure undergoes significant conformational changes upon binding to the ligand, differing from the structure employed for docking (see Figure 3). These results highlight their potential to enhance the accuracy of pose sampling in protein-ligand docking applications.

### 4.2 Comparing IVES and GLOW to flexible protein docking

We compared the performance of GLOW and IVES with the open-source Smina flexible protein docking (“Smina flexible”), specifically focusing on cross-docking cases where “Smina flexible” completed within 48 hours on a single CPU, constituting 40% of the dataset (see Figure S2 for details). Despite this selective assessment, GLOW and IVES consistently outperformed “Smina flexible,” especially in challenging and AlphaFold benchmarks (Figure S3). Using 20 protein conformations, GLOW and IVES demonstrated significantly faster runtimes—approximately 20 minutes and 6–7 hours, respectively—on a single CPU compared to “Smina flexible,” which typically required 16 hours. While IVES’s runtime scales linearly with the number of protein conformations in a serialized setting, it exhibits high parallelizability, completing in around 20 minutes on average when fully parallelized. Moreover, IVES offers extensive customization, allowing users to adjust the sampling process by specifying the number of protein conformations and the maximum number of generated poses per docking run for each conformation. This flexibility enables a balance between thoroughness and computational costs.

IVES also achieved comparable sampling performance to Schrodinger IFD-MD, a proprietary state-of-the-art flexible protein docking software, using only 20 protein conformations versus IFD-MD’s 1000 conformations (Figure S5). Notably, IVES—unlike IFD-MD—does not rely on an experimentally co-determined ligand pose in the docking protein structure, which allows IVES to work with unliganded or predicted structures such as those from AlphaFold, broadening its applicability.

Overall, our results not only highlight the competitive sampling capabilities of GLOW and IVES, which in some cases outperformed flexible protein docking, but also underscores their value in scenarios where access to experimentally determined ligand poses within the docking protein structure is unavailable.

## 5 Discussion

Our benchmark results demonstrate the substantial improvements achieved by GLOW and IVES in increasing the probability of sampling correct poses in protein-ligand docking. These gains are especially notable in challenging and AlphaFold benchmarks, where protein structures exhibit significant conformational differences when bound to query ligands compared to those used in docking. Additionally, IVES generates multiple protein conformations, which can be beneficial for geometric deep learning on protein structures. Furthermore, we provide datasets of candidate ligand poses generated by our methods for approximately 5,000 protein-ligand cross-docking pairs. These datasets may serve as valuable resources for developing and assessing deep-learning-based scoring functions in molecular docking.

While IVES demonstrates the best performance, its sampling efficiency is contingent upon the quality of the initial seed poses. Ideally, these initial poses should closely resemble experimentally determined poses to reduce the need for generating numerous protein conformations during sampling, as illustrated in Figure S6. The selection of these poses relies on an effective scoring function, creating a complex interplay between scoring and sampling. Nevertheless, IVES offers a customizable workflow for ligand pose sampling, allowing the generation of improved samples to train a machine-learned protein-ligand docking scoring function. This scoring function, in turn, can refine seed pose selection in IVES, establishing a dynamic feedback loop that continuously improves pose sampling and scoring accuracy.

## Supplementary Material

1

## References

[R1] Allen William J, Balius Trent E, Mukherjee Sudipto, Brozell Scott R, Moustakas Demetri T, Lang P Therese, Case David A, Kuntz Irwin D, and Rizzo Robert C. Dock 6: Impact of new features and current docking performance. Journal of computational chemistry, 36(15):1132–1156, 2015. 25914306 10.1002/jcc.23905 PMC4469538

[R2] Bajusz D£vid, Rlcz Anita, and Hberger Kroly. Why is tanimoto index an appropriate choice for fingerprint-based similarity calculations? Journal of cheminformatics, 7(1):1–13, 2015. 26052348 10.1186/s13321-015-0069-3 PMC4456712

[R3] Bender Brian J, Gahbauer Stefan, Luttens Andreas, Lyu Jiankun, Webb Chase M, Stein Reed M, Fink Elissa A, Balius Trent E, Carlsson Jens, Irwin John J, A practical guide to large-scale docking. Nature protocols, 16(10):4799–4832, 2021. 34561691 10.1038/s41596-021-00597-z PMC8522653

[R4] Cole Jason C, Murray Christopher W, Nissink J Willem M, Taylor Richard D, and Taylor Robin. Comparing protein-ligand docking programs is difficult. Proteins: Structure, Function, and Bioinformatics, 60(3):325–332, 2005. 10.1002/prot.20497 15937897

[R5] Eastman Peter, Swails Jason, Chodera John D, McGibbon Robert T, Zhao Yutong, Beauchamp Kyle A, Wang Lee-Ping, Simmonett Andrew C, Harrigan Matthew P, Stern Chaya D, Openmm 7: Rapid development of high performance algorithms for molecular dynamics. PLoS computational biology, 13(7):e1005659, 2017. 28746339 10.1371/journal.pcbi.1005659 PMC5549999

[R6] Ferreira Leonardo G, Dos Santos Ricardo N, Oliva Glaucius, and Andricopulo Adriano D. Molecular docking and structure-based drug design strategies. Molecules, 20(7):13384–13421, 2015. 26205061 10.3390/molecules200713384 PMC6332083

[R7] Forli Stefano, Huey Ruth, Pique Michael E, Sanner Michel F, Goodsell David S, and Olson Arthur J. Computational protein-ligand docking and virtual drug screening with the autodock suite. Nature protocols, 11(5):905–919, 2016. 27077332 10.1038/nprot.2016.051 PMC4868550

[R8] Francoeur Paul G, Masuda Tomohide, Sunseri Jocelyn, Jia Andrew, Iovanisci Richard B, Snyder Ian, and Koes David R. Three-dimensional convolutional neural networks and a cross-docked data set for structure-based drug design. Journal of chemical information and modeling, 60(9):4200–4215, 2020. 32865404 10.1021/acs.jcim.0c00411 PMC8902699

[R9] Friesner Richard A, Banks Jay L, Murphy Robert B, Halgren Thomas A, Klicic Jasna J, Mainz Daniel T, Repasky Matthew P, Knoll Eric H, Shelley Mee, Perry Jason K, Glide: a new approach for rapid, accurate docking and scoring. 1. method and assessment of docking accuracy. Journal of medicinal chemistry, 47(7):1739–1749, 2004. 15027865 10.1021/jm0306430

[R10] Jones Gareth, Willett Peter, Glen Robert C, Leach Andrew R, and Taylor Robin. Development and validation of a genetic algorithm for flexible docking. Journal of molecular biology, 267(3): 727–748, 1997. 9126849 10.1006/jmbi.1996.0897

[R11] Jordan John B, Whittington Douglas A, Bartberger Michael D, Sickmier E Allen, Chen Kui, Cheng Yuan, and Judd Ted. Fragment-linking approach using 19f nmr spectroscopy to obtain highly potent and selective inhibitors of ,0-secretase. Journal of Medicinal Chemistry, 59(8):3732–3749, 2016. 26978477 10.1021/acs.jmedchem.5b01917

[R12] Jumper John, Evans Richard, Pritzel Alexander, Green Tim, Figurnov Michael, Ronneberger Olaf, Tunyasuvunakool Kathryn, Bates Russ, Zfdek Augustin, Potapenko Anna, Highly accurate protein structure prediction with alphafold. Nature, 596(7873):583–589, 2021. 34265844 10.1038/s41586-021-03819-2 PMC8371605

[R13] Kitchen Douglas B, Decornez H6lfene, Furr John R, and Bajorath Jurgen. Docking and scoring in virtual screening for drug discovery: methods and applications. Nature reviews Drug discovery, 3 (11):935–949, 2004. 15520816 10.1038/nrd1549

[R14] Koes David Ryan, Baumgartner Matthew P, and Camacho Carlos J. Lessons learned in empirical scoring with smina from the csar 2011 benchmarking exercise. Journal of chemical information and modeling, 53(8):1893–1904, 2013. 23379370 10.1021/ci300604z PMC3726561

[R15] Kontoyianni Maria, McClellan Laura M, and Sokol Glenn S. Evaluation of docking performance: comparative data on docking algorithms. Journal of medicinal chemistry, 47(3):558–565, 2004. 14736237 10.1021/jm0302997

[R16] KrishnaRohith, WangJue, AhernWoody, SturmfelsPascal, VenkateshPreetham, KalvetIndrek, LeeGyu Rie, Morey-BurrowsFelix S, AnishchenkoIvan, HumphreysIan R, Generalized biomolecular modeling and design with rosettafold all-atom. bioRxiv, pages 2023–10, 2023.10.1126/science.adl252838452047

[R17] Lemmon Gordon and Meiler Jens. Rosetta ligand docking with flexible xml protocols. Computational Drug Discovery and Design, pages 143–155, 2012. 10.1007/978-1-61779-465-0_10 PMC3749076 22183535

[R18] Liu Zhihai, Li Yan, Han Li, Li Jie, Liu Jie, Zhao Zhixiong, Nie Wei, Liu Yuchen, and Wang Renxiao. Pdb-wide collection of binding data: current status of the pdbbind database. Bioinformatics, 31(3): 405–412, 2015. 25301850 10.1093/bioinformatics/btu626

[R19] Miller Edward B, Murphy Robert B, Sindhikara Daniel, Borrelli Kenneth W, Grisewood Matthew J, Ranalli Fabio, Dixon Steven L, Jerome Steven, Boyles Nicholas A, Day Tyler, Reliable and accurate solution to the induced fit docking problem for protein-ligand binding. Journal of Chemical Theory and Computation, 17(4):2630–2639, 2021. 33779166 10.1021/acs.jctc.1c00136

[R20] Paggi Joseph M, Belk Julia A, Hollingsworth Scott A, Villanueva Nicolas, Powers Alexander S, Clark Mary J, Chemparathy Augustine G, Tynan Jonathan E, Lau Thomas K, Sunahara Roger K, Leveraging nonstructural data to predict structures and affinities of protein-ligand complexes. Proceedings of the National Academy of Sciences, 118(51):e2112621118, 2021. 10.1073/pnas.2112621118 PMC8713799 34921117

[R21] Park Heuisul, Min Kyeongsik, Kwak Hyo-Shin, Ki Dong Koo Dongchul Lim, Seo Sang-Won, Choi Jae-Ung, Platt Bettina, and Choi Deog-Young. Synthesis, sar, and x-ray structure of human bace-1 inhibitors with cyclic urea derivatives. Bioorganic & medicinal chemistry letters, 18(9): 2900–2904, 2008. 18434152 10.1016/j.bmcl.2008.03.081

[R22] Ravindranath Pradeep Anand, Forli Stefano, Goodsell David S, Olson Arthur J, and Sanner Michel F. Autodockfr: advances in protein-ligand docking with explicitly specified binding site flexibility. PLoS computational biology, 11(12):e1004586, 2015. 26629955 10.1371/journal.pcbi.1004586 PMC4667975

[R23] Shen Chao, Ding Junjie, Wang Zhe, Cao Dongsheng, Ding Xiaoqin, and Hou Tingjun. From machine learning to deep learning: Advances in scoring functions for protein-ligand docking. Wiley Interdisciplinary Reviews: Computational Molecular Science, 10(1):e1429, 2020.

[R24] Shen Chao, Zhang Xujun, Deng Yafeng, Gao Junbo, Wang Dong, Xu Lei, Pan Peichen, Hou Tingjun, and Kang Yu. Boosting protein-ligand binding pose prediction and virtual screening based on residue-atom distance likelihood potential and graph transformer. Journal of Medicinal Chemistry, 65(15):10691–10706, 2022. 35917397 10.1021/acs.jmedchem.2c00991

[R25] Sherman Woody, Day Tyler, Jacobson Matthew P, Friesner Richard A, and Farid Ramy. Novel procedure for modeling ligand/receptor induced fit effects. Journal of medicinal chemistry, 49(2): 534–553, 2006. 16420040 10.1021/jm050540c

[R26] Suriana Patricia, Paggi Joseph M, and Dror Ron O. Flexvdw: A machine learning approach to account for protein flexibility in ligand docking. arXiv preprint arXiv:2303.11494, 2023.

[R27] Varadi Mihaly, Anyango Stephen, Deshpande Mandar, Nair Sreenath, Natassia Cindy, Yordanova Galabina, Yuan David, Stroe Oana, Wood Gemma, Laydon Agata, Alphafold protein structure database: massively expanding the structural coverage of protein-sequence space with high-accuracy models. Nucleic acids research, 50(D1):D439–D444, 2022. 34791371 10.1093/nar/gkab1061 PMC8728224

[R28] Verdonk Marcel L, Cole Jason C, Hartshorn Michael J, Murray Christopher W, and Taylor Richard D. Improved protein-ligand docking using gold. Proteins: Structure, Function, and Bioinformatics, 52(4):609–623, 2003. 10.1002/prot.10465 12910460

[R29] Wang Renxiao, Fang Xueliang, Lu Yipin, Yang Chao-Yie, and Wang Shaomeng. The pdbbind database: methodologies and updates. Journal of medicinal chemistry, 48(12):4111–4119, 2005. 15943484 10.1021/jm048957q

