# Supplementary material for: Enhancing Ligand Pose Sampling for Molecular Docking
Source: ArXiv. 2023 Nov 30:arXiv:2312.00191v1. Preprint. [Version 1] (PMC10705564)
Supplement: 1 [file NIHPP2312.00191v1-supplement-1.pdf]

Supplementary Information

S1 Datasets

To create ligand pose datasets from the PDBBind 2019 refined dataset [Wang et al., 2005, Liu et al., 2015], we initially categorized protein-ligand complex structures based on the protein, grouping structures of the same target protein. To ensure a balanced representation in our dataset and prevent bias from overrepresented proteins with numerous experimentally determined structures bound to various ligands, we randomly selected a maximum of 15 structures for each protein to serve as docking protein structures. Furthermore, the selection of protein-ligand cross-docking pairs took into account the Tanimoto similarity between the query ligand to be docked and the co-determined ligand present in the docking structure [Bajusz et al., 2015], with a threshold of less than 0.4, indicating dissimilarity. For each unique ligand, we chose up to 5 protein structures as cross-docking pairs. This resulted in 4,102 distinct protein-ligand cross-docking pairs (derived from 238 unique proteins), ensuring diversity and relevance in our dataset.

As outlined in 3.2, the "challenging (AlphaFold)" dataset includes the same cross-docking pairs as "challenging (experimental)," but it employs AlphaFold protein structures for docking, in contrast to experimentally determined ones. However, the "challenging (experimental)" dataset contains cross-docking pairs with structures from the same protein but bound to different ligands, often referred to as "holo structures." Consequently, the "challenging (AlphaFold)" dataset has fewer cross-docking pairs available when using AlphaFold structures for docking, as those holo structures of the same protein will be mapped to the same AlphaFold structure.

All the datasets contain poses generated through GLOW and IVES. We followed the protocols described in Paggi et al. [2021] for preparing protein-ligand complex structures and ligands prior to the docking process. IVES produced poses using 5 protein conformations in a single iteration, generating a maximum of 300 poses for each docking to each protein conformation. Seed poses are selected based on the RTMscore [Shen et al., 2022].

S2 Docking settings

In Figure 3, all pose sampling methods were run with the following settings: an exhaustiveness value of 16, a minimum RMSD filter set at 1.5Å and a search space box sized at 20Å with its center aligned to the bound ligand pose within the docking protein structure. In cases involving AlphaFold-generated structures, the search space center was determined using an experimentally determined ligand pose (not the query ligand). In practical scenarios where experimentally resolved ligand poses are unavailable, search space determination can be facilitated using binding pocket finder tools. Additionally, to create softened VDW potentials for GLOW and IVES, we adjusted the repulsion weight to 0.2, departing from the default value of 0.840.

S3 Distributions of the number of sampled poses across different methods

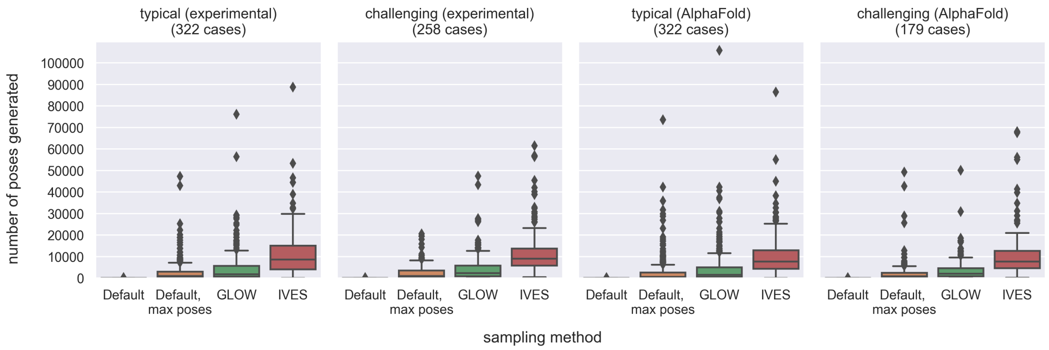

Figure S1: Distribution of the number of poses generated by GLOW and IVES compared to baseline methods "Default" and "Default, max poses". To ensure a fair comparison between IVES, GLOW, and "Default, max poses," we allowed "Default, max poses" and "GLOW" to generate as many poses as possible (specifically, up to 1 million poses per ligand for "Default, max poses" and up to an additional 1 million poses per ligand with the softened VDW potential in GLOW). Nevertheless, IVES typically generated more poses than these other methods, because IVES utilizes multiple protein conformations, expanding the feasible pose landscape.

S4 Comparison of GLOW and IVES sampling performances with Smina flexible protein docking

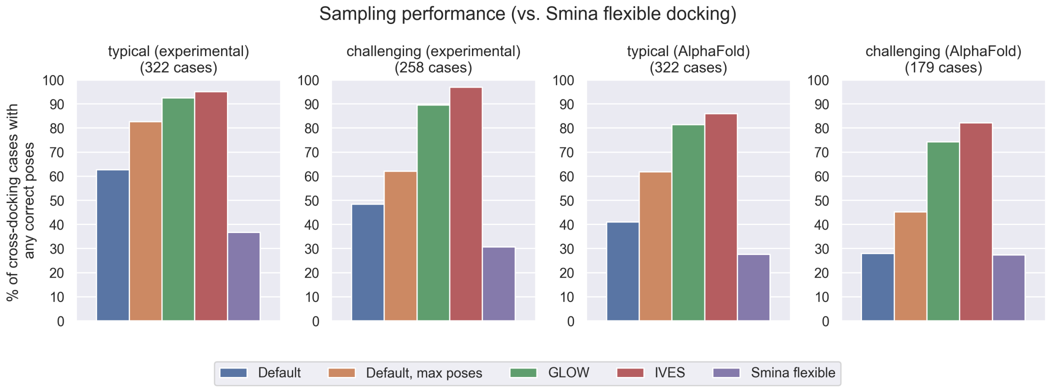

Figure S2: Sampling performance of GLOW and IVES compared to Smina flexible protein docking on the test sets, measured by the percentage of cross-docking cases with at least one correct pose. In this analysis, we compare the performance of the GLOW and IVES, as described in Figure 3, with Smina flexible protein docking ("Smina flexible"). Similar to Figure 3, "Smina flexible" is run with a search space box of size 20Å centered around the bound ligand pose in the protein structure used for docking. We aim to achieve a similar pose count for "Smina flexible" whenever possible. It's important to note that "Smina flexible" operates under a 48-hour time limit, with runs exceeding this duration considered failures. Among the results, 20% of "Smina flexible" runs did not complete within 48 hours, 40% completed within the timeframe but failed to generate poses, while the remaining 40% completed within 48 hours and successfully generated poses. These statistics collectively contribute to the relatively inferior performance of "Smina flexible" compared to other methods, including the baseline approaches "Default" and "Default, max poses."

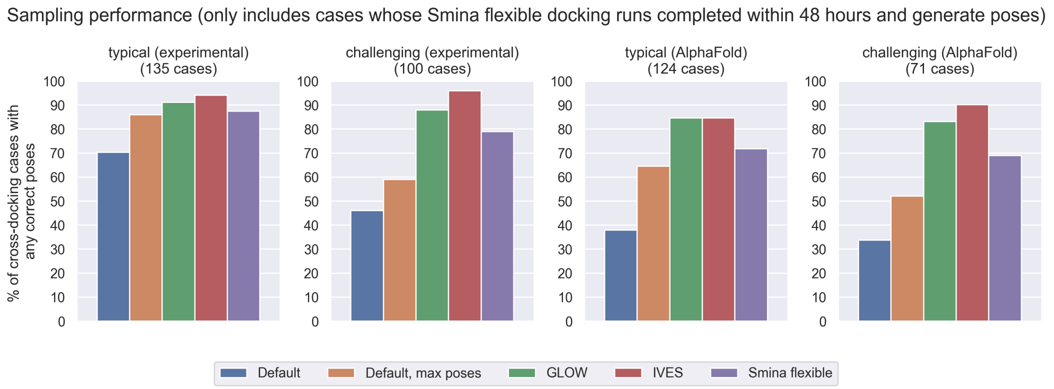

Figure S3: Sampling performance of GLOW and IVES compared to Smina flexible protein docking across multiple datasets, measured by the percentage of cross-docking cases with at least one correct pose, focusing on cases where "Smina flexible" completed within 48 hours on one CPU and generated poses. This analysis differs from that of Figure S3 in that we only consider cross-docking cases where "Smina flexible" successfully completed within 48 hours on one CPU and generated poses, accounting for approximately 40% of the total cases. Even in this subset, both GLOW and IVES consistently outperform "Smina flexible", particularly in challenging and AlphaFold benchmarks where the protein structure undergoes substantial conformational changes upon binding to the ligand, differing from the structure employed during the docking process. In addition, GLOW and IVES are considerably faster than Smina flexible protein docking. On average, GLOW finishes in about 20 minutes, while IVES typically takes 6-7 hours on a single CPU. In contrast, "Smina flexible" runs, completed within a 48-hour timeframe, average around 16 hours. It's worth highlighting IVES' high parallelizability, achieving an average completion time of approximately 20 minutes when fully parallelized. Furthermore, IVES offers extensive customization options, allowing users to adjust sampling thoroughness by selecting the number of protein conformations or setting the maximum number of generated poses per docking with each conformation. This flexibility empowers users to strike a balance between thoroughness and computational costs.

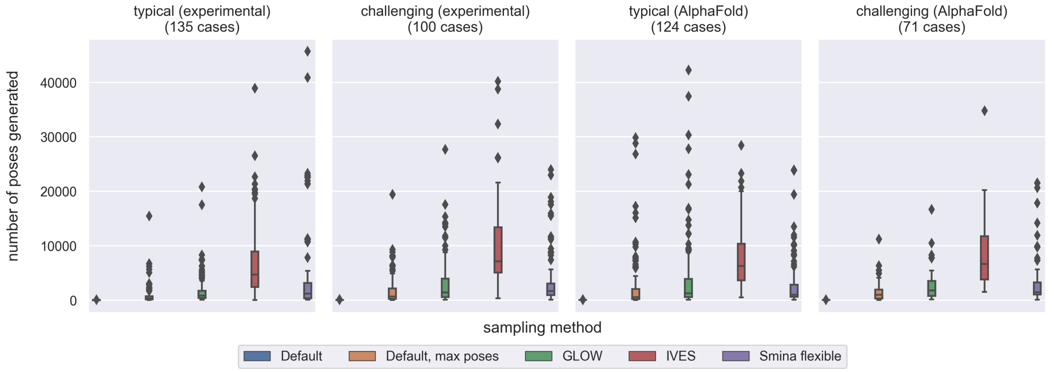

Figure S4: Distribution of the number of poses generated by GLOW and IVES compared to Smina flexible docking ("Smina flexible") and baseline methods "Default" and "Default, max poses". Here, we only consider cross-docking cases where "Smina flexible" successfully completed within 48 hours on one CPU and generated poses, accounting for approximately 40% of the total cases. To ensure a fair comparison, we allowed "Default, max poses" and "GLOW" to generate as many poses as possible.

S5 Comparison of IVES sampling performance with Schrödinger IFD-MD flexible protein docking

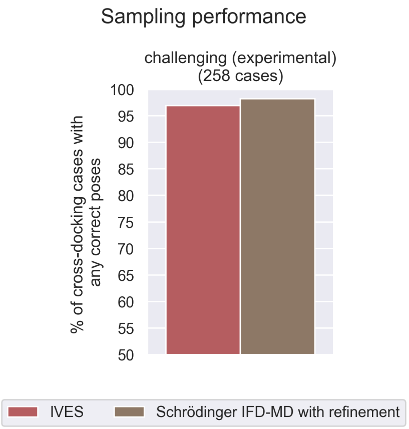

Figure S5: Sampling performance of IVES vs. Schrödinger IFD-MD on "challenging (experimental)" dataset. Here we compare the sampling performance (measured by the percentage of cross-docking cases with at least one correct pose) of IVES versus Schrödinger IFD-MD (with refinement). IVES (in red) exhibited comparable performance to Schrödinger IFD-MD (in brown), despite using only 20 protein conformations compared to IFD-MD's 1000. Notably, IVES doesn't rely on an experimentally co-determined ligand pose bound in the docking structure, making it applicable to docking to unliganded or predicted structures such as those generated by AlphaFold.

S6 The impact of the number of protein conformations and seed pose quality on IVES sampling performance

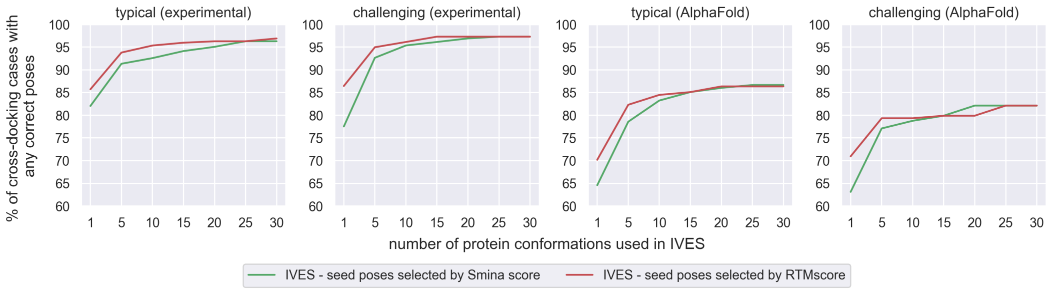

Figure S6: Sampling performance of IVES as measured by the percentage of cross-docking cases with at least one correct pose as a function of the number of protein conformations used for sampling. As we increase the number of protein conformations employed by IVES, we observe a significant increase in the percentage of cross-docking cases yielding correct poses. This increase is most noticeable when using 1 to 5 protein conformations. It's important to note that IVES runtime scales proportionally with the number of protein conformations, but its high parallelizability efficiently utilizes computational resources. Therefore, for those with computational constraints, running IVES with 5 protein conformations strikes a favorable balance between resources and sampling performance. Efficient IVES sampling relies on the quality of seed poses chosen for generating protein conformations. Better seed poses, ideally close to the "correct" pose, reduce the need for large number of protein conformations, thus lowering computational demands. We employ both the Smina docking score and RTMscore [Shen et al., 2022], a machine-learned scoring function for ranking ligand poses, to rank and select seed poses. In our evaluation, RTMscore emerges as the better choice for ranking, enhancing IVES's sampling efficiency with fewer protein conformations compared to when using Smina docking score (highlighted in red vs. green). This emphasizes the critical role of seed pose quality in optimizing IVES's sampling outcomes.
